# Properties and Mechanisms of Deletions, Insertions, and Substitutions in the Evolutionary History of SARS-CoV-2

**DOI:** 10.3390/ijms25073696

**Published:** 2024-03-26

**Authors:** Igor B. Rogozin, Andreu Saura, Eugenia Poliakov, Anastassia Bykova, Abiel Roche-Lima, Youri I. Pavlov, Vyacheslav Yurchenko

**Affiliations:** 1 Life Science Research Centre, Faculty of Science, University of Ostrava, 710 00 Ostrava, Czech Republic; 2 National Eye Institute, National Institutes of Health, Bethesda, MD 20892, USA; 3 Center for Collaborative Research in Health Disparities—RCMI Program, Medical Sciences Campus, University of Puerto Rico, San Juan 00936, Puerto Rico; 4 Eppley Institute for Research in Cancer and Allied Diseases, University of Nebraska Medical Center, Omaha, NE 68198, USA

**Keywords:** SARS-CoV-2, epistasis, mutation hotspots, ADAR, APOBEC, oxidative stress, viral fitness, low-complexity regions

## Abstract

SARS-CoV-2 has accumulated many mutations since its emergence in late 2019. Nucleotide substitutions leading to amino acid replacements constitute the primary material for natural selection. Insertions, deletions, and substitutions appear to be critical for coronavirus’s macro- and microevolution. Understanding the molecular mechanisms of mutations in the mutational hotspots (positions, loci with recurrent mutations, and nucleotide context) is important for disentangling roles of mutagenesis and selection. In the SARS-CoV-2 genome, deletions and insertions are frequently associated with repetitive sequences, whereas C>U substitutions are often surrounded by nucleotides resembling the APOBEC mutable motifs. We describe various approaches to mutation spectra analyses, including the context features of RNAs that are likely to be involved in the generation of recurrent mutations. We also discuss the interplay between mutations and natural selection as a complex evolutionary trend. The substantial variability and complexity of pipelines for the reconstruction of mutations and the huge number of genomic sequences are major problems for the analyses of mutations in the SARS-CoV-2 genome. As a solution, we advocate for the development of a centralized database of predicted mutations, which needs to be updated on a regular basis.

## 1. Introduction

Mutations are generally classified as induced (caused by exposure to exogenous mutagenic factors) or spontaneous (occurring in the absence of such an exposure). Mutagenesis in vivo is a complex multi-step process involving DNA/RNA molecules and enzymes involved in DNA/RNA precursor metabolism, DNA/RNA replication, recombination, and repair [1,2,3]. The process of mutation is an essential and fundamental evolutionary factor, which creates genetic variation. Spontaneous mutagenesis is a result of inaccuracies in the replication of genomic material [4]. The factors that determine mutation rate and specificity are now more amenable to analysis as more data on mutation distributions (mutation spectra) become available [5]. A mutation spectrum is a distribution of frequencies of mutations along the nucleotide sequence of a reference genome (for example, and relevant to this work, SARS-CoV-2 Wuhan-Hu-1, GenBank ID NC_045512). The most frequently used source of these data is computational reconstructions of mutations in sets of aligned sequences [6,7,8,9,10]. Another source of mutational spectra is experimental test systems. A good example of this is a delineated set of recurrent deletions acquired in the N-terminal domain of the SARS-CoV-2 spike glycoprotein, which alter defined antibody epitopes during long-term infections in cancer patients [11].

In this paper, we discuss deletions, insertions, and substitutions in the SARS-CoV-2 genome. We describe various approaches to mutation spectra analysis, including the context features of RNA that give rise to mutation “hotspots”. This pattern is different from those of influenza B RNA viruses, whose evolution is primarily driven by reassortments and insertions–deletions [12,13]. Of note, writing a review paper on this topic is a very challenging task, primarily because of the overwhelming number of SARS-CoV-2-related papers that have been published. For instance, the PubMed database indexed approximately 4700 relevant papers published in just 3 months between 1 January 2020 and 12 April 2020 [14]. 

## 2. Results

### 2.1. SARS-CoV-2 Genome Structure and Replication

**Figure 1:**
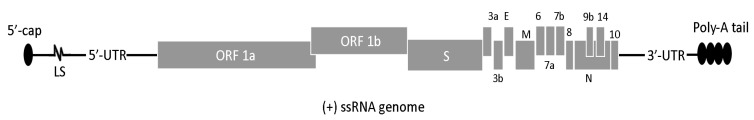
Structure of the SARS-CoV-2 genome. The 5′-cap, UTR sequences, leader sequences (LSs), poly-A tail, and standard names of ORFs are shown. M, N, S, and E are structural proteins.

The SARS-CoV-2 genome is a positive-sense single-stranded RNA molecule, about 30 kb in length, with the typical gene organization of coronaviruses [15,16]. There are a dozen functional or putatively functional ORFs that encode over 25 proteins, including 16 non-structural proteins (NSP1 to NSP16), four structural proteins (M, N, S, and E), and several accessory proteins, including ORF3a, ORF3b, ORF6, ORF7a, ORF7b, and ORF8 (Figure 1). Accessory proteins are not essential for replication in cell culture. However, they may play regulatory roles during the viral cycle in the host cells and, thus, contribute to the virus’s fitness by increasing its ability to modify the host’s immune response [17,18]. Coronaviruses usually differ in which of these accessory proteins they possess, and more infective species often have specific virulent features associated with these proteins [19]. A recent study suggested that the coding capacity of SARS-CoV-2 is likely to have been underestimated. A high-resolution map of protein-coding regions in the SARS-CoV-2 genome revealed 23 previously unannotated viral ORFs [20]. The exact number of functional ORFs in the SARS-CoV-2 genome is being debated, as can be exemplified by ORF10, the functionality of whose protein product has been questioned [21].

Recurrent replication is an essential step in the viral lifestyle. The RNA-dependent RNA polymerase (NSP12) of the SARS-CoV-2 virus is error prone, with many errors being corrected by the proofreading activity of the 3′-to-5′ exoribonuclease (NSP14) [17,18,22]. Coronaviruses lacking exoribonuclease activity are susceptible to lethal mutagenesis because the rate of mutations increases from ~10^−6^/bases per infection cycle to ~10^−5^/bases per infection cycle [23].

Viruses in the family *Coronaviridae* (order *Nidovirales*) replicate through the transcription of negative-sense RNA intermediates that serve as templates for positive-sense genomic RNA, and an array of sub-genomic RNAs that are generated from discontinuous transcription during the synthesis of negative-strand RNA. Template switching occurs at transcription-regulating sequences (TRSs) located at the 5′ UTRs of the leader sequences and the TRSs located upstream of various genes in the distal third of the genome [24,25,26]. This process produces sub-genomic RNAs that contain a 5′ UTR leader sequence (labeled LS in Figure 1), which are fused to the sequence derived from one of the downstream genes. It is highly likely that a high abundance of sub-genomic RNAs at the 5′ and 3′ ends of the viral genome creates various biases in the distributions and frequencies of mutations across the genomic sequence.

### 2.2. Reconstructions and Analyses of Mutation Spectra: Methodological Approaches

**Figure 2:**
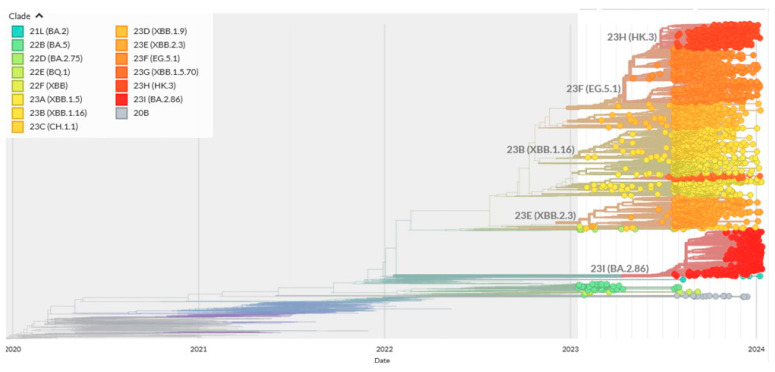
Typical Nextstrain tree with a detailed resolution for the January 2023—January 2024 time period. In total, 3213 out of 3972 sequences sampled between January 2023 and January 2024 have been used to reconstruct the tree by Nextstrain. Different colors on the phylogenetic tree correspond to names of SARS-CoV-2 strains shown at the upper left panel.

As mentioned above, important sources of mutation spectra are computational reconstructions of mutations using variability data across sets of the aligned-to-the-reference SARS-CoV-2 sequences. However, sequencing errors in low-quality sequences and errors in bioinformatics pipelines can potentially produce high rates of false positives. Thus, the quality of sequencing is a very important issue. The vast majority of sequences used in this study were obtained using nanopore technology, which is not always accurate in regions with low coverage. Because many closely related sequences are produced by the same sequencing center, this tendency is likely to cause systemic biases. While all current analysis pipelines are designed to eliminate spurious mutations [7], the sheer number of sequenced SARS-CoV-2 genomes (see, for example, the Nextstrain system [27]) makes this task extremely challenging. An example of a phylogenetic tree reconstructed by the Nextstrain online system for a limited number of sequences (usually less than 4000) is shown in the Figure 2. 

There are two main approaches to delineate viral mutations. The simplest one is to count the mutations at a given position on a SARS-CoV-2 sequence alignment and assume that they emerged only once [28,29]. A heuristic threshold for the minimum number of mutations to be observed at a given position is set by the researcher. The obvious pitfall of this approach is frequently missing recurrent mutations, reversals (backward mutations), and indels (insertions and deletions). However, the approach is useful for analyses of long insertions and deletions [28,30,31]. A substantially more sophisticated approach for the prediction of mutations is based on phylogenetic inferences [7] and allows detection of recurrent mutations and reversals. Some positions/regions (called mutational hotspots) have a high frequency of recurrent mutations, suggesting that they may be under episodic positive selection [9]. 

Phylogenetic trees (i.e., Figure 2) can be inferred using various methods, yet all of them have certain limitations. As an example, the least squares distance and maximum parsimony approaches to predicting deletions in over 600 thousand SARS-CoV-2 genomes produced many false positive hits [30]. Inaccuracies of phylogenetic reconstructions and the difficulty of predicating the ancestral sequences that are used to infer mutations are well known [32,33]. Maximum likelihood estimation techniques and Bayesian approaches for tree reconstructions and the prediction of ancestral sequences usually tend to produce better results than those based on parsimony and distances [6,7,8,9,10,27]. However, the sample sizes for such inferences should be reasonably small because the phylogenetic models used are highly complex. The pipelines for mutation reconstructions rely on numerous assumptions. In a recent paper [7], the authors used a pre-built clade-annotated UShER (Ultrafast Sample placement on Existing tRee [34]) mutation-annotated tree from the UCSC website and matUtils [35] to place a subset of the mutation-annotated trees on the samples from each Nextstrain clade (Figure 2) and then to extract the mutations for each branch [7]. Next, they tallied the counts for each mutation on all the branches for a given clade, manually excluding sites that are likely to be prone to errors due to abnormally large numbers of mutations [7]. This step was necessary considering that many recurrent mutations in the reported SARS-CoV-2 genome sequences have been observed predominantly or exclusively by single labs; moreover, they co-localize with annealing sites for the commonly used primers and are more likely to affect the protein-coding sequences than other similarly recurrent mutations [33]. 

**Figure 3:**
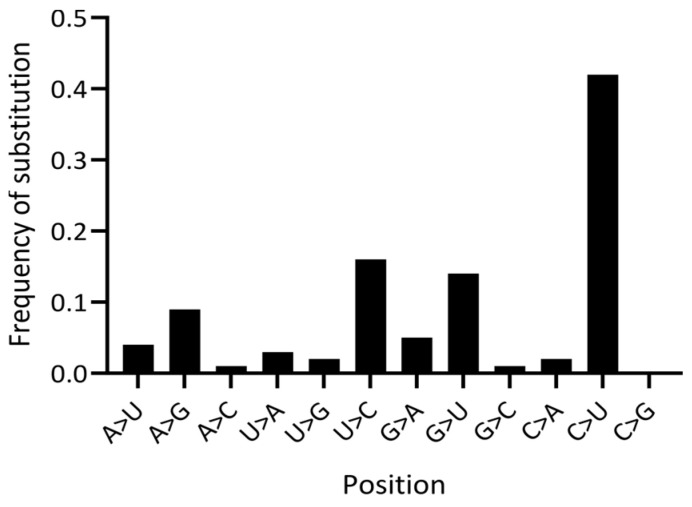
Substitution frequencies in SARS-CoV-2. The Y axis is the fraction of each predicted mutation type in 4-fold degenerate sites. Data are from [9].

The analytical approaches presented above are instrumental in understanding the role of mutational hotspots, prediction of recurrent mutations, and context analysis [6,7,8,36]. Statistical analysis of the mechanisms of mutations and selection is an important part of SARS-CoV-2 studies. The simplest approach to studying mutational spectra is to analyze the frequencies of substitutions. An example of such an analysis is shown in Figure 3. 

**Figure 4:**
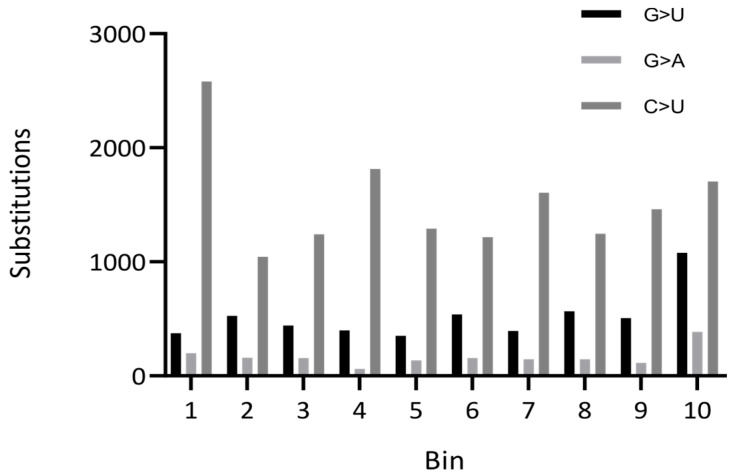
Distribution of mutations across coding regions of the SARS-CoV-2 genome. The number of substitutions is shown for each of the 10 equal-length bins in the viral genome. Data are from [9].

The analysis of distributions of mutations and frameshift and non-frameshift deletions or insertions across the SARS-CoV-2 genome is another useful tool for analyses. An example of a distribution of substitutions across the SARS-CoV-2 genome is presented in Figure 4. 

Studies of three-dimensional (3D) structures of proteins can be an exceptionally informative approach to infer their functions. For SARS-CoV-2, the most frequently analyzed protein is spike, although some other proteins have been investigated in this regard too [37,38,39,40,41]. An example of a successful study using the 3D approach is an analysis of ORF8, which is a rapidly evolving accessory protein thought to interfere with immune responses [37,38,39,40,41]. The 3D structure of SARS-CoV-2 ORF8 was determined by X-ray crystallography. The structure revealed a ∼60-residue core sequence homologous to SARS-CoV-2 ORF7a, with an addition of two dimerization interfaces unique to SARS-CoV-2 ORF8 [37,38,39,40,41]. The presence of these interfaces suggested that SARS-CoV-2 ORF8 is able to form unique protein assemblies that are not possible for SARS-CoV ORF8. These assemblies are likely to mediate unique immune suppression and evasion activities [37,38,39,40,41].

Analysis of nonsynonymous and synonymous substitutions is used to analyze the modes of natural selection and trends in the evolution of protein-coding genes [42,43,44]. The Ka/Ks (the ratio of the rate of nonsynonymous nucleotide substitutions, which lead to a change in the encoded amino acid, to the rate of synonymous ones) are commonly used to distinguish between purifying and positive selection. Ka/Ks below one reflect purifying selection, whereas Ka/Ks over one may indicate positive (Darwinian) selection. Among synonymous substitutions, the four-fold degenerate sites (sites at the third position on codons, where all three possible nucleotide mutations are synonymous) and non-coding RNA regions are expected to be the best approximation of nearly neutral modes of evolution [42,43,44]. 

Published sets of SARS-CoV-2 sequences, reconstructed phylogenetic trees, and predicted mutations are available from a variety of databases (Table 1). It should be noted that these datasets are the results of computational studies and are not always supported for long periods of time. For example, the CoV-GLUE database is not regularly updated, at least not for deletions in SARS-CoV-2. The GESS database was last updated in March 2023. This is understandable considering the overwhelming amount of SARS-CoV-2 raw sequences. We think that the next important step is to develop comprehensive datasets of predicted mutations that will contain the information on putative recurrent mutations and reversions exemplified by the recent databases UShER [34] and CoVigator [45]. This is an extremely challenging task considering the major problems discussed above; however, the absence a centralized database of predicted mutations is hindering further analysis of the mechanisms of mutations and trends in the evolution of SARS-CoV-2. 

### 2.3. Molecular Mechanisms of Mutations

SARS-CoV-2 has accumulated many mutations during the several years of the pandemic [36]. Mutations leading to amino acid substitutions constitute the primary raw material for genetic variation; however, many insertions, deletions, and recombination events are likely to be critical elements in the macro- and microevolution of coronavirus [30,46,47]. Understanding the molecular mechanisms of mutations is important in itself, but it is also essential for understanding the role of mutation hotspots and uncovering the pathways of their appearance. For example, an increased frequency of deletions in the genes encoding the ORF6-ORF7a-ORF7b-ORF8 (Figure 1) complex of accessory proteins in SARS-CoV-2 is likely due to the fact that these genes evolve under the forces of natural selection [30,47,48].

Mutational changes in DNA/RNA molecules are classified into point mutations and large-scale recombination events. Point mutations are substitutions, deletions, and insertions. An additional class is rare complex mutations, which are various combinations of the types of mutations mentioned above. It is generally accepted that point mutations represent a mutation process; for example, errors of RNA replication or RNA repair. However, there is no clear-cut border between these classes of events, as, for example, gene conversion between partially homologous sequences may also result in point mutations [3,49]. Mutational hotspots are frequently associated with the context of the surrounding sequences, such as RNA secondary structure, presence of homonucleotide sequences, direct and inverted repeats, minisatellites, short mutable motifs, and other DNA sequence features.

#### 2.3.1. Deletions

**Figure 5:**
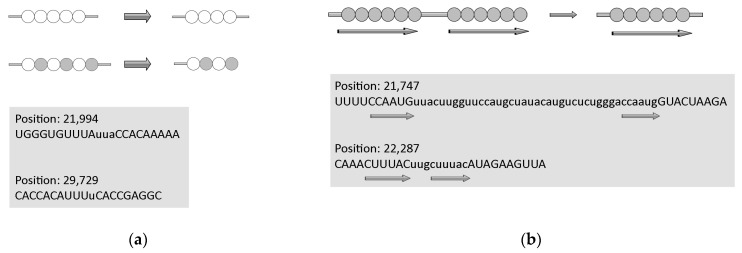
Molecular mechanisms of deletions in the SARS-CoV-2 genome. (**a**) Template dislocation model for short deletions: one (or several) nucleotide deletions in short stretches of identical nucleotides or polynucleotides. (**b**) Template switch model for long deletions: deletion between direct repeats that includes removal of one repeat. Lowercase letters indicate deleted regions, direct repeats are shown by arrows. Data are from [30]. Circles correspond to nucleotides, empty and filled circles are used depending on the nature of repetitive sequences.

Repeated RNA/DNA sequences are prone to various RNA/DNA rearrangements. The removal of one or both copies of repeated sequences is the result of so-called illegitimate recombination. These rearrangements depend on the close proximity of the repeated sequences and can occur between direct repeats ranging from several to hundreds of nucleotides [50,51,52]. We have to mention that all these studies on DNA have been conducted in bacteria. It has been proposed that these non-recombinational rearrangements may occur via a template dislocation (Figure 5a) or a template switch misalignment (Figure 5b) of the repeated sequences during RNA replication. The importance of deletions at repeated sequences is widely recognized because these events (for example, deletions/duplications of trinucleotide repeat arrays) are responsible for many genetic diseases in humans [53].

Short deletions are well-known to be associated with stretches of identical nucleotides or tandemly arranged di- and tri-nucleotides (low-complexity regions, Figure 5a). This tendency was also documented for single-nucleotide deletions in the SARS-CoV-2 genome. For example, the numbers of deletions in stretches of two identical nucleotides are similar to those of deletions in stretches of three and four identical nucleotides, although the observed numbers of identical stretches in the SARS-CoV-2 genome are dramatically different. This strongly indicates that many short deletions are the results of so-called template dislocation in stretches of identical nucleotides (Figure 5b), which likely emerged from RNA polymerase errors [30]. An important feature of short deletions in SARS-CoV-2 is a substantial excess of these events in UTRs compared to the coding regions, implying that, to a large extent, deletions in coding regions are true deletion events rather than just sequencing errors. It is quite likely that short deletions in stretches of identical nucleotides may occur independently in different viral lineages. Some short deletions are supported by anecdotal observations. For example, the UUA deletion (Figure 5a) is one of the mutation signatures of the highly infectious B.1.1.7 lineage that accounted for many COVID-19 cases [54].

Long deletions are less likely to emerge independently many times. Many long deletions are flanked by short direct repeats with zero or one–two mismatches, suggesting template switching (a variant of illegitimate recombination) as the main mechanism of deletions [30]. A more complex scenario of the interplay between deletions and insertions simulated by inverted repeats in single stranded RNA has been recently proposed for several SARS-CoV-2 genes [55]. Indeed, the hairpins formed by inverted repeats have long been known to be associated with deletions and elevated intra- and inter-chromosomal recombination [56,57]. 

#### 2.3.2. Insertions

**Figure 6:**
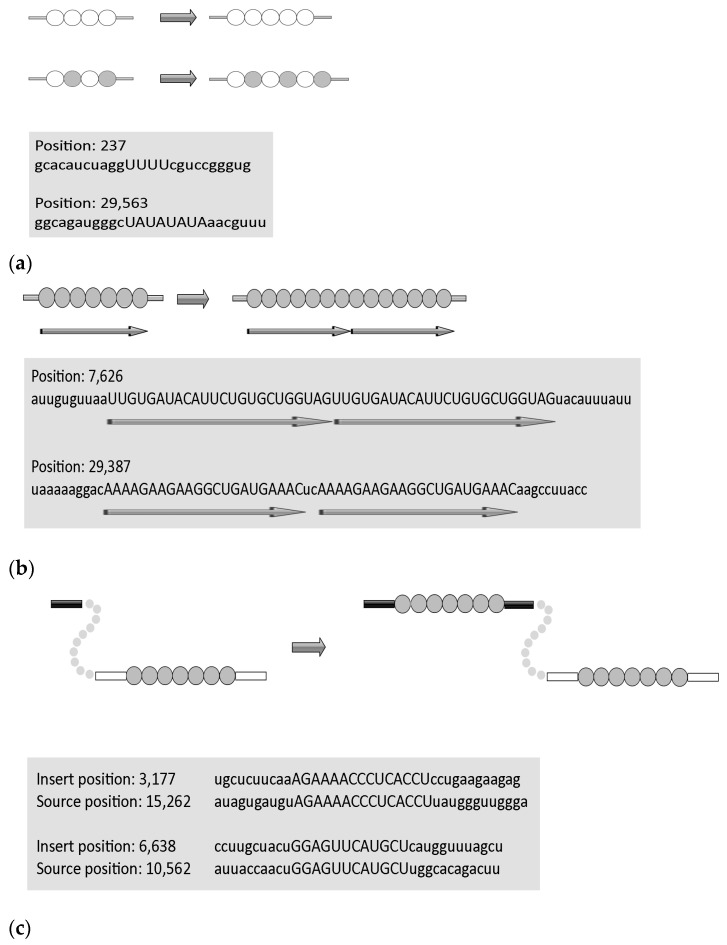
Molecular mechanisms of insertions in the SARS-CoV-2 genome. (**a**) Template dislocation model: one (or several) nucleotide insertions in short stretches of identical nucleotides or polynucleotides. Example of short insertions. (**b**) Duplications. (**c**) Template switch model for long insertions. Lowercase letters indicate flanking regions. Data are from [28]. Circles correspond to nucleotides, empty and filled circles are used depending on the nature of repetitive sequences.

Similar to deletions, short insertions also tend to be associated with stretches of identical nucleotides or tandemly arranged di- and tri-nucleotides [28]. They were strongly enriched in Us and, in most cases, emerged independently (as judged by phylogenetic inferences). It is most parsimonious to suggest that these insertions resulted from RNA-dependent RNA polymerase (RdRp) slippage on short runs of A or U (Figure 6a). In contrast, the composition of the long insertions (Figure 6b) was close to that of the SARS-CoV-2 genome, and many of these insertions were found to be monophyletic; that is, these appear to be rare events that did not occur on nucleotide runs. It should be noted that many long insertions have been manually created, in some cases using long-read nanopore sequencing. Sequence analysis of the SARS-CoV-2 genomes indicates that these insertions occur either through polymerase slippage resulting in tandem duplication or, more commonly, illegitimate template switching (Figure 6c) associated with the formation of sgRNAs [28]. In support of the latter hypothesis, template switching in different RNA viruses (including coronaviruses) has been demonstrated previously in a variety of experimental settings. For approximately one third of the long insertions, the authors were not able to pinpoint the source of the inserted sequence. One possible explanation is a mutational deterioration between the source and the inserted sequences, especially for relatively short insertions, but another unknown mechanism of illegitimate recombination cannot be ruled out [28].

#### 2.3.3. Substitutions

Transitions (C<->T(U) and A<->G mutations) tend to be overrepresented in the spectra of spontaneous mutations (so-called transition bias) [58] and favored over transversions (C<->A, C<->G, T(U)<->A, T(U)<->G) [59,60]. Transition bias has been clearly recognized as a general property of DNA/RNA-sequence evolution, having been observed in all types of genomes in prokaryotes, eukaryotes, and viruses [61,62,63,64].

For SARS-CoV-2, a large proportion of the substitutions are likely to be caused by the RdRp transcription errors incorporated during replication. These mutations are expected to be approximately symmetrical (for example, C>U and G>A mutations should have similar frequencies [36]). In other words, a tendency to mis-incorporate a U instead of a C would, therefore, be reflected in a parallel number of G>A mutations occurring on the minus strand. However, the frequency of G>A mutations in the SARS-CoV-2 genome was substantially lower than that of C>U, and generally comparable to the transitions of A>G and U>C (Figure 3) [65]. It has been proposed that an excess of C>U mutations in SARS-CoV-2 is caused by the activity of the host APOBEC (cytosine deaminases) family of RNA editing enzymes [29,36,66]. Indeed, the APOBECs deaminate C to U in single-stranded nucleic acids and function in a variety of biological processes, including innate and adaptive immune responses to viral pathogens [67]. Members of the APOBEC3 family are reported to be involved in the control of DNA and RNA viruses [68]. While most APOBECs use single-stranded DNA (ssDNA) as a substrate for cytosine deamination, three APOBECs (APOBEC1, APOBEC3A, and APOBEC3G) deaminate certain cellular single-stranded RNA (ssRNA) targets [69]. Experimental data suggest that APOBEC3A is likely be involved in C>U mutagenesis in SARS-CoV-2 [70]. As for the A>G transitions, they can be caused by the action of ADAR (Adenosine Deaminase Acting on RNA) RNA editing enzymes [71], although no obvious excess of A>G and U>C mutations was detected in the mutational spectra of SARS-CoV-2 (Figure 3).

Another unusual property of the SARS-CoV-2 genome is an apparent excess of G>U transversions (Figure 3) [29,72]. One possible explanation for these data is the unusual properties of the SARS-CoV-2 replication machinery. However, this would be an exceptionally rare evolutionary phenomenon—just the second of its kind along with an exonuclease-deficient four-subunit DNA polymerase epsilon complex of *Saccharomyces cerevisiae* [73]. Another possible explanation is oxidative mutagenesis generating 8-oxoG in viral RNA [74,75,76]. Replication of 8-oxoG with the insertion of A would be manifested as a G>U mutation in the strand where 8-oxoG was present [29]. Distribution analysis of G>U and C>U mutations across the SARS-CoV-2 genome suggests that distributions are not Gaussian, with elevated frequencies at the 3′ and 5′ ends of the alignment, respectively (Figure 4). Thus, the mechanisms of C>U and G>U mutations are likely to be different.

### 2.4. Natural Selection of Mutations

The nucleic acids of rapidly evolving pathogens are subject to the strongest evolutionary forces that have been reported in evolutionary biology [77]. A good example of this is the evolution of the antigenic variation of African trypanosomes with variant surface glycoprotein genes, which are under selection pressure in adapting to their hosts’ defenses [78,79]. Viruses too frequently undergo adaptive changes at genomic sites that are targeted by immune responses [80,81,82]. However, many mutations experience dramatic changes in frequencies across the whole viral population in a matter of months or even weeks [83]. Although most mutations are effectively neutral, or even negatively affect viral fitness, a small number of them emerge and spread in viral populations, suggesting a positive effect on viral fitness and adaptive evolution [9].

#### 2.4.1. Selection of Deletions and Insertions

Analysis of in-frame and out-of-frame deletions and insertions detected a significant excess of in-frame mutations [36]. In-frame deletions are expected to have lesser functional consequences compared to out-of-frame deletions. Single nucleotide deletions are relatively frequent, with a substantial fraction of them occurring in ORF6, ORF7a, ORF7b, and ORF8 genes (Figure 1) [30]. The indels are likely to affect the antigenic properties of SARS-CoV-2. For example, a 382-nucleotide deletion in the ORF8 found in several genotypes was correlated with a milder infectivity [48]. Recent evidence has established the presence of recurrent deletion regions that map to the defined antibody epitopes. As such, recurrent deletions in the N-terminal domain of the S glycoprotein can alter the defined antibody epitopes during long-term infections of immunocompromised patients [11]. Insertions are also unevenly distributed along the SARS-CoV-2 genome. For instance, all seven insertions in the spike glycoprotein localize to its N-terminal domain (NTD) [28]. This domain attracts much of researchers’ attention now because it has been shown to harbor multiple substitutions associated with SARS-CoV-2 variants of concern and those detected in immunocompromised individuals with long COVID-19 [84,85,86]. 

**Figure 7:**
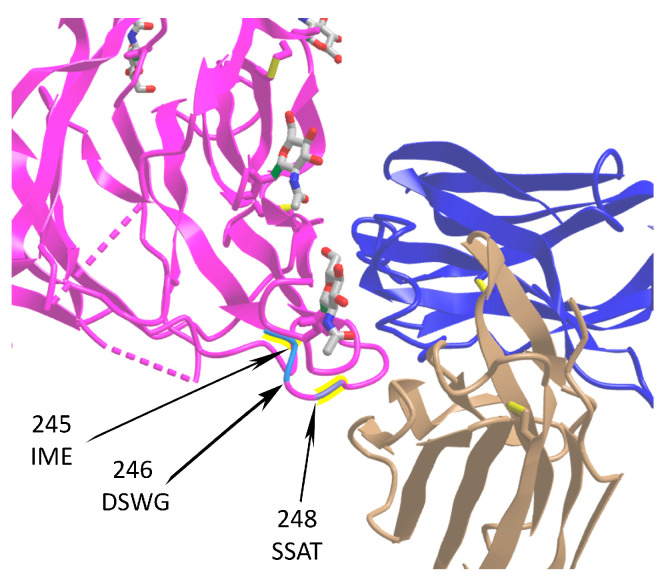
Location of three insertion sites in the SARS-CoV-2 S protein affecting spike–IgV (immunoglobulin variable domain) binding surfaces. The spike protein is shown in magenta (PDB ID: 7cn8), while light (PDB ID: 7cl2) and heavy (PDB ID: 7cl2) chains of 4A8 antibody are in beige and blue, respectively. Sequences of insertions at positions 245, 246, and 248 are shown. The data are taken from [28]. The monosaccharide N-acetylglucosamine (NAG) molecules are shown at the surface of spike.

All high-confidence insertions in the spike glycoprotein mentioned above have been located on the protein’s surface, with three of them overlapping with the recently described antibody epitope [87], making them potentially involved in the virus’s immune escape (Figure 7). An important feature of short and long indels in SARS-CoV-2 is their substantial excess of UTRs compared to coding regions [30]. It has been hypothesized that the increased frequency of indels, their non-random distribution, and their independent co-occurrence in several lineages, are the potential mechanisms of viral responses to the elevated immunity of the global population [30,36].

#### 2.4.2. Selection of Substitutions

The evolution of SARS-CoV-2 during the pandemic was primarily driven by purifying selection (0.1 < Ka/Ks < 0.5), but a small set of sites (such as the receptor-binding domain (RBD) on the spike protein and the region of the nucleocapsid protein determining nuclear localization) appear to evolve under positive selection [9,88]. The most highly constrained sequences corresponded to some NSPs and the M protein. Conversely, genes encoding NSP1 and accessory ORFs (Figure 1), particularly ORF8, had substantial proportions of codons evolving under conditions of very weak purifying (close to neutral) selection [88]. The six bona fide positively selected sites were located on the N protein, ORF8, and NSP1. A signal of positive selection was also detected in the RBD of the S protein, but it most likely resulted from a recombination event that involved the BatCoV RaTG13 sequence [88]. In line with previous data, it was suggested that the common ancestor of SARS-CoV-2 and BatCoV RaTG13 encoded/encodes an RBD similar to that of SARS-CoV-2 and some pangolin viruses [88].

### 2.5. Interplay between Mutations and Selection

Successful transmission to new hosts requires numerous adaptive changes, such as receptor specificity adjustment in the coronavirus itself or to the longer-term evolutionary arms race with the host’s antiviral defense system [89,90]. Initial escape mutations almost invariably carry a fitness cost but are frequently compensated for by subsequent fitness-restoring mutations [9,38,91]. A sizable fraction of amino acid substitutions appears to be fixed by positive selection, but it is unclear to what degree long-term protein evolution is constrained by epistasis; that is, instances when substitutions that are accepted in one genotype are deleterious in another [92]. 

For SARS-CoV-2, it has been suggested that a small set of sites evolves under positive selection. These sites form a strongly connected network of apparent epistatic interactions and are signatures of major clades in the SARS-CoV-2 phylogeny. Multiple mutations, some of which have since been demonstrated to enable antibody evasion, began to emerge in association with ongoing regional diversification, indicating the emergence of new SARS-CoV-2 strains [9]. Another interesting example is the numerous nonsynonymous mutations acquired in the Omicron lineage before it became the most frequent variant of SARS-CoV-2 [38,93]. Relative to the original Wuhan-Hu-1 strain, this variant has approximately 37 mutations in the spike protein that is responsible for binding and entry into host cells. Fifteen of them are in the RBD that binds to the host’s angiotensin-converting enzyme 2 (ACE2) receptor and serves as a target for many neutralizing antibodies. This structure of the spike protein when bound to human ACE2 provides a rationale for the observed evasion of antibodies elicited by previous vaccinations or infections and shows how mutations that weaken ACE2 binding are compensated for by mutations that enable new interactions [40,41]. All these results indicate that the evolution of the Omicron spike protein is driven to a large extent by epistatic interactions.

There is also an apparent link between a particular deletion and natural selection in the SARS-CoV genome. Among the most dramatic genomic changes observed in SARS-CoV isolated from patients during the peak of the pandemic in 2003 was the acquisition of a characteristic 29-nucleotide deletion in ORF8 causing its split into two smaller ORFs, ORF8a and ORF8b (Figure 1) [94]. Functional consequences of this event were not entirely clear, but recent evolutionary analyses of ORF8a and ORF8b genes suggested that they are under purifying selection, thus proteins translated from these ORFs are likely to be functionally important [31]. 

### 2.6. A Puzzle: Insertion and Recurrent Deletions of the -PRRA- Sequence

**Figure 8:**
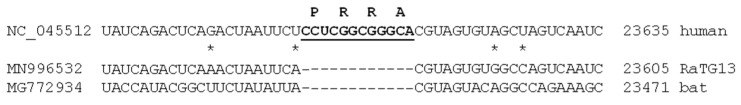
Sequences surrounding the CCTCGGCGGGCA insertion in the SARS-CoV-2 sequence. MN996532 is the closest bat homolog RaTG13; MG772934 is a more distantly related bat homolog. Asterisks indicate mismatches between SARS-CoV-2 and RaTG13. Letters above NC_045512 correspond to encoded amino acids.

In its early evolution, the SARS-CoV-2 spike glycoprotein acquired a new four amino acid -PRRA- insertion at positions 681–684 (encoded by -CCU CGG CGG GCA- at the RNA level) (Figure 8) [95,96]. This sequence is absent from all other known bCoV lineages, such as SARS-CoV and MERS-CoV [95,96]. It formed a novel furin cleavage site in the S protein [97]. This is significant because furin protease is abundant in the respiratory tract and found throughout the body. It is also “employed” by other RNA viruses, including HIV, influenza, dengue, and Ebola virus, to enter the cell. Conversely, the proteases typically used by SARS-CoV are much less abundant and widespread, and not as effective. Although the virus probably gained the insertion through an as yet unknown illegitimate recombination event, this particular furin site sequence has never been found in any other coronavirus from any other species [98]. The functional consequences of the -PRRA- insertion at the RNA level (Figure 8) are not well understood. However, the translation of viral RNA depends on various factors. It has been suggested that this insertion may have a cumulative effect by providing both furin cleavage and translation pausing sites, allowing the virus to infect its new host (humans) more readily [98]. This underlines the importance of ribosome pausing for the efficient regulation of protein translation and, also, of co-translational subdomain folding, as suggested by experimental studies [98]. 

**Figure 9:**
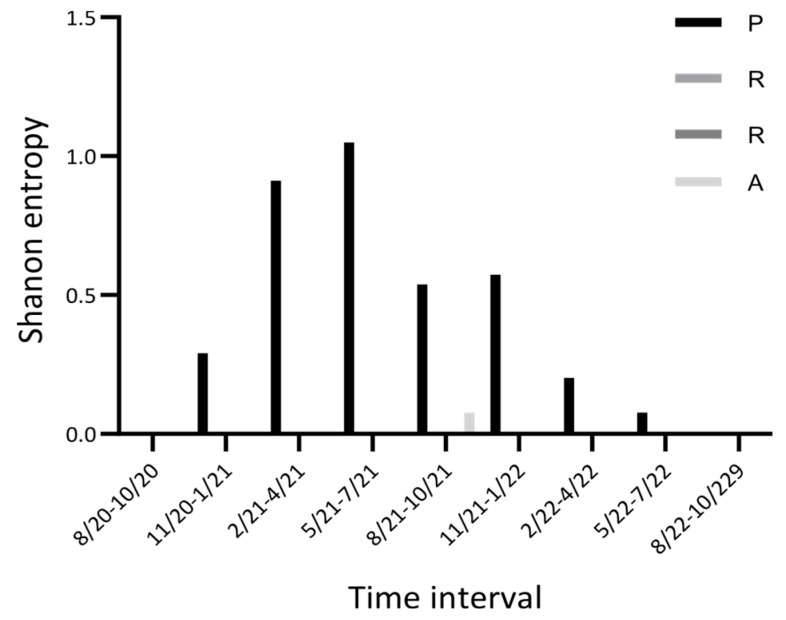
Time-series plot of the Nextstrain entropy (a normalized Shannon entropy) for the PRRA/HRRA/LRRA inserted sequences.

The initial -PRRA- has subsequently transformed into the -HRRA- or -LRRA- sequence [99]. The functional consequences of these mutations are not entirely clear. It is parsimonious to suggest that the -HRRA- variant impacts the infectivity, pathogenesis, and transmissibility of the virus [40,99,100]. The dynamics of the normalized Shannon entropy of the first position of -PRRA- appear dramatic; virtually no variability was detected for the July–October 2020 and July–October 2022 periods, whereas a substantial increase followed by a dramatic decrease of variability was documented between November 2020 and June 2022 (Figure 9). The last three positions of the -PRRA- sequence did not vary. 

Notably, a deletion of the furin recognition site and neighboring regions on the spike gene has been detected in a substantial fraction of sub-genomic viral RNAs [101]. Deep sequencing and ribosomal profiling data showed that the fraction of this genomic deletion was small (~2%) in the early stages of viral infection. However, this fraction is likely to increase in the late stages of infection, diminishing its potential role in the S protein’s expression [20]. The functional consequences of this “reversion” to the ancestral state are not clear and certainly warrant further studies, as it may reflect on one of the key mechanisms of successful reproduction of SARS-CoV-2 in human cells.

## 3. Discussion

Various approaches have been developed to infer mutations in the SARS-CoV-2 genome. However, the field would definitely benefit from a centralized database of mutations, which must be updated on a regular basis. This will make it easier to find and correct the shortcomings of various approaches and improve the quality of the dataset in a systematic way. For example, recurring biases in tree reconstructions may create substantial problems in downstream analysis [32,33]. This becomes especially important when considering the controversial and contradictory results that can be found in the literature. For example, a study from 2020 documented a substantial excess of A>G and U>C mutations in eight patients, reporting that the fraction of C>U mutations was smaller in comparison and detecting no excess of G>U [102]. These observations (made on a small number of samples) contradict later studies, although one must bear in mind that subsequent studies reported on data collected in the later stages of pandemics [7,65]. 

The role and impact of APOBECs and ADARs in inducing a high rate of C>U mutations is not entirely clear. There is experimental evidence that supports this hypothesis [70], making computational predictions more credible. Another challenge is to understand the mechanisms of G>U mutations. Whether they are driven by oxidative damage generating 8-oxoG in viral RNA [29,76], or a different mechanism [7], remains to be investigated. This is important in light of a recent observation of changes in G>U transversion frequency over time (the relative rate of these mutations in the Omicron variant is about two times lower than in early clades of SARS-CoV-2 [7]).

We believe that any computational prediction must be thoroughly validated experimentally. However, this is not as straightforward as it appears because of the extremely high transmissibility of SARS-CoV-2. In vitro experiments with RdRp can help to estimate the error rates and understand the context specificities of mutations. Similar experiments can be informative when combined with computational studies. For example, a computational RNA context analysis suggested that APOBECs can play a prominent role in SARS-CoV-2 mutagenesis. This prediction was tested in cell culture, which confirmed that APOBEC1, APOBEC3A, and APOBEC3G can edit the specific sites of SARS-CoV-2 RNA which cause C>U mutations during viral RNA replication. Interestingly, SARS-CoV-2 replication and progeny production in Caco-2 cells were not inhibited by overexpression of these APOBECs. Instead, overexpression of APOBEC3A promoted viral replication and propagation, implying that APOBEC-mediated mutations are likely to cause changes in fitness and potentially influence the evolution of SARS-CoV-2 [70]. Another example of a successful combination of computation predictions and experimental studies is an investigation of deletions in the ORF7a gene. Several ORF7a deletions of different sizes (190, 339, and 365 nt) have been identified in COVID-19-positive patients with mild symptoms. Computational analyses suggested that the deletions impair ORF7a function. While isolated viruses with deleted ORF7a can replicate similarly to the wild-type viruses in vitro, they produce fewer infectious particles [103]. These findings contribute to our understanding of SARS-CoV-2 replication and immune evasion, as well as providing insights into the role of ORF7a in virus–host interactions. These results are consistent with the recent observation that ORF7a is a hotspot of long deletions in the SARS-CoV-2 genome [30].

Studying the dynamics of mutations in various groups of COVID-19 patients is another promising avenue of research. Analyses of SARS-CoV-2 microevolution in immunocompromised patients confirmed recurrent deletions in the N-terminal domain of the S glycoprotein that are likely to alter defined antibody epitopes during long-term infections of these patients [11]. Further studies of SARS-CoV-2 genomic sequences in patients experiencing different symptoms and clinical outcomes will provide additional information to increase our understanding of the mechanisms of mutations and the role of natural selection in viral evolution. The analysis of different geographical locations and populations can also provide new information about the properties of viral mutations. It has been found that some samples from Africa have a significantly higher frequency of substitutions compared to those from other geographical locations [104]. Furthermore, comparative analyses of the virus in various human tissues can help us to understand trends of viral evolution. It is well-known that ACE2 (angiotensin-converting enzyme 2) is the primary receptor that mediates infections in human cells [105]. However, it has been suggested that SARS-CoV-2 infections in several types of human cells are primarily mediated by LDLRs (low-density lipoprotein receptors) [106,107]. Further experimental analyses of various strains of SARS-CoV-2 may uncover the molecular mechanisms and dynamics of these crucial interactions. 

Previous studies of SARS-CoV and MERS-CoV provided a significant amount of information about various aspects of coronaviral evolution and functioning within host species. Numerous interspecies transmission events were detected for both viruses [108]; however, SARS-CoV-2 studies brought many new observations. This is expected because of an unprecedented joint effort among many scientists from all over the world. Although the origin of the SARS-CoV-2 infection in humans remains unknown, infections have been frequently reported in different animal species. At least fifteen species are known to have been positive for the Delta variant and ten species have been documented as being infected with two different types of viral variants, suggesting human-to-animal, animal-to-animal, and animal-to-human transmission events [109]. Mutations play a crucial role in these processes, as exemplified by the -PRRA- insertion.

In conclusion, computational and experimental studies of mutations are useful for gaining a deep understanding of trends in mutagenesis and natural selection. Even small changes in the structure of SARS-CoV-2 genes can substantially affect fitness and the trajectories of viral evolution. Analyses of these trends echo those of cancer mutations in humans and some other mammalian species. However, centralized databases of cancer mutations and related information are updated on a regular basis, predicted mutational signatures and mutable motifs are constantly refined, RNA/DNA contexts have been specified for predictions and analyses of cancer driver mutations, and many individual mutational signatures have been studied experimentally [5]. We are confident that further computational and functional analyses of mutations in SARS-CoV-2 genomes will be able to draw on similar resources in the near future.

## Figures and Tables

**Table 1:** Frequently used online SARS-CoV-2 resources (all accessed on 23 January 2024).

Database	Link
CDC strains	https://www.cdc.gov/coronavirus/2019-ncov/variants
NCBI	https://www.ncbi.nlm.nih.gov/activ
Nextstrain	https://nextstrain.org/ncov/gisaid/global/6m
GISAID	https://gisaid.org
SARS-CoV-2 mutation portal	http://sarscov2-mutation-portal.urv.cat
CoV-GLUE	https://cov-glue.cvr.gla.ac.uk
UShER	https://genome.ucsc.edu/cgi-bin/hgPhyloPlace
GESS	https://wan-bioinfo.shinyapps.io/GESS/
CoVigator	https://github.com/TRON-bioinformatics/covigator

## Data Availability

Publicly available datasets were analyzed in this study. These data can be found at the following online resources: https://www.ncbi.nlm.nih.gov/activ; https://gisaid.org (accessed on 23 January 2024); http://sarscov2-mutation-portal.urv.cat; https://cov-glue.cvr.gla.ac.uk (accessed on 23 January 2024); https://www.cdc.gov/coronavirus/2019-ncov/variants (accessed on 23 January 2024); https://nextstrain.org/ncov/gisaid/global/6m (accessed on 23 January 2024); https://genome.ucsc.edu/cgi-bin/hgPhyloPlace (accessed on 23 January 2024); https://wan-bioinfo.shinyapps.io/GESS/ (accessed on 23 January 2024); https://github.com/TRON-bioinformatics/covigator (accessed on 23 January 2024).
